# Responses of small mammals to habitat characteristics in Southern Carpathian forests

**DOI:** 10.1038/s41598-021-91488-6

**Published:** 2021-06-08

**Authors:** Ana Maria Benedek, Ioan Sîrbu, Anamaria Lazăr

**Affiliations:** 1 Faculty of Sciences, Lucian Blaga University of Sibiu, 5-7 Raţiu Street, 550012 Sibiu, Romania; 2 Department of Engineering and Management in Food and Tourism, Faculty of Food and Tourism, Transilvania University of Brașov, 148 Castelului Street, 500036 Braşov, Romania

**Keywords:** Ecology, Zoology, Ecology

## Abstract

Compared to Northern Carpathians, the small mammal fauna of Southern Carpathian forests is poorly known, with no data on habitat use; our study seeks to fill this gap. To this end, we conducted a survey in the Southern Carpathians for five years, assessing habitat use by small mammals in forests along an elevational gradient. Trapping was done using live traps set in transects at elevations between 820 and 2040 m. For each transect we evaluated variables related to vegetation structure, habitat complexity, and geographical location. We considered abundance, species composition and species richness as response variables. The rodents *Apodemus flavicollis* and *Myodes glareolus* and the shrew *Sorex araneus* were common and dominant. Their abundance were positively correlated with tree cover, the best explanatory variable. Responses to other variables were mixed. The strong divergence in the relative habitat use by the three most abundant species may act as a mechanism that enables their coexistence as dominant species, exploiting the same wide range of habitat resources. Overall, habitat use in our study area was similar to that reported from Northern Carpathians, but we found also important differences probably caused by the differences in latitude and forest management practices.

## Introduction

The Southern Carpathian Mountains, which comprise about one-third of Romania, are heavily forested, with beech (51.8%) and spruce (33.6%) being the dominant species^1^. Unlike in western and central Europe, where intensive forest management has resulted in even-aged monocultures, especially of spruce and other conifers, forest management in Romania is not so intensive and thus the natural composition of tree species is usually preserved. Most of Romania’s natural forests are in the mountains.

Small mammals are key components of these forest ecosystems, where they have multiple functions, such as acting as seed and fungus dispersers^2–4^ and soil aerators^5^. Meantime, they exercise direct and indirect top-down and bottom-up control on the distribution, abundance, and population dynamics of other animal taxa^6,7^ by being important predators of insects and other invertebrates^8^ and food resources for many vertebrate predators. On the other hand, seed predation and consumption of tree bark by rodents can have negative effects on forests, with young trees being especially affected^9^, thus potentially hindering forest regeneration^10^.

The presence and abundance of small mammals in forests are often correlated with habitat characteristics^11,12^, one of the most important being vegetation cover. Tree canopy cover often is negatively correlated with density and complexity of understory vegetation^13^, influencing the availability of resources on the forest floor. Thus, abundance of small mammals is often positively correlated with forests of thin or sparse canopy^14^. Habitat complexity, including vegetation heterogeneity and the presence and abundance of logging debris usually increase availability of food and shelter, promoting reproduction and/or survival of several species of small mammals, resulting in higher species richness and total abundance^15^.

Although several studies have evaluated the effects of habitat characteristics and management on the structure in small mammal communities in forests of the Northern Carpathians^16,17^, the few studies in the Southern Carpathians have been mainly faunistic (i.e., lists of species), although a habitat description is sometimes also provided. None have examined the responses of small mammal species to habitat characteristics, as we report here. We use data from a survey of small mammal communities along elevational gradients in forests with various habitat characteristics at 26 trapping sites in the Southern Carpathian Mountains in protected and unprotected areas.

Most often the response to habitat characteristics varies greatly among the species of an ecological community and some species may show different responses even to human disturbance^18^. But habitat selection may change also within a species along geographical gradients^19^, in accordance with the physiological and behavioural traits^20^ acquired in time under certain environmental conditions, so that the habitat preferences observed in some part of the species’ range cannot be used to predict habitat use elsewhere. Therefore, the knowledge of specific and detailed responses of small mammals to habitat factors enables taking the adequate forest management measures, either for enhancing diversity and abundance of rare species or to reduce abundance of troublesome species, e.g., to control forest damage.

Our small mammal survey sought to assess habitat use of small mammals along an elevational gradient. We evaluated the responses of small mammals to characteristics of montane forest habitats, at both community and species levels. Thus, we analysed relationships among several habitat characteristics that describe vegetation structure, habitat complexity, and habitat location (elevation and distance to the closest watercourse) as explanatory variables, and species richness, species abundance, total abundance, community abundance, and species composition of small mammals as response variables. We also tested the effect of human disturbance by logging (in the unprotected area) and tourism (in the Retezat National Park) on these parameters.

## Material and methods

### Description of the study area

**Figure 1:**
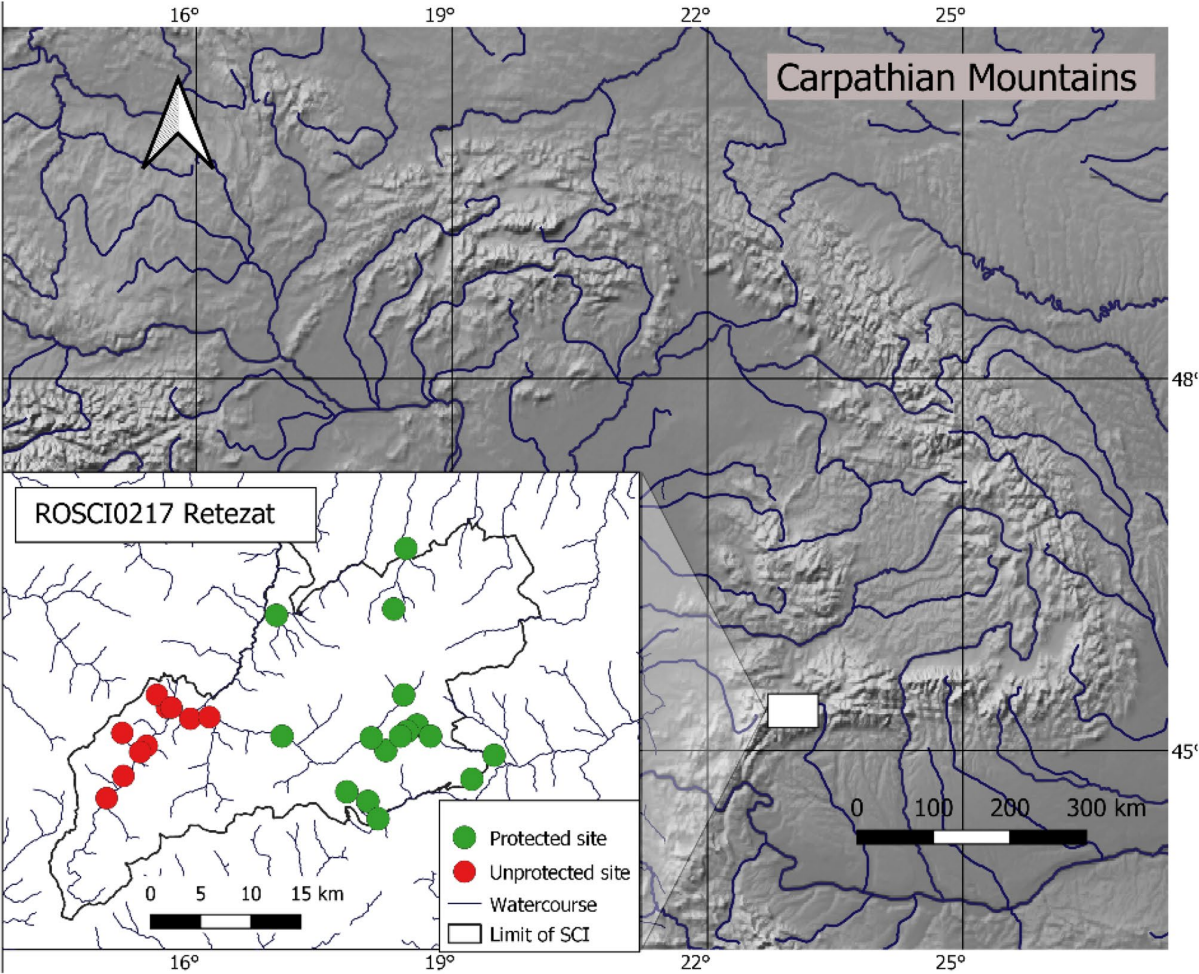
Location of the 26 trapping sites along elevational gradients (820–2040 m) in the Southern Carpathian Mountains of Romania in central Europe. SCI stands for Site of Community Interest, a protected area part of the European Natura 2000 network. The map was made in QGIS^21^ with a base map from Natural Earth^22^.

We conducted our study in the Retezat, Țarcu and Godeanu massifs of the Southern Carpathian Mountains, Romania. Most sampling sites were located in the Retezat National Park (the oldest protected area in Romania, established in 1935) and the others in an adjacent area (not protected at the time of survey). Both areas are presently included in the ROSCI0217 Retezat (Fig. 1), part of the Natura 2000 European network of protected areas. This protected area was established in 2009, after the completion of our field work. Most of the study area is a mix of virgin, natural and planted forests. The natural elevational succession of forests in the study area (and throughout the Southern Carpathians) starts with beech (*Fagus sylvatica*) forests at lower elevations (SI, Fig. A1a), followed by mixed forests composed of differing proportions of beech and Norway spruce (*Picea abies*) with scattered silver fir (*Abies alba*) and sycamore (*Acer pseudoplatanus*) (SI, Fig. A1b). A Norway spruce forest belt reaches up to the timberline, which usually is present at elevations between 1600 and 1800 m, depending on slope, exposition and other geomorphological characteristics of the site. The shrub and herbaceous layers in spruce forests is composed mainly of spruce saplings (SI, Fig. A1c). The tree canopy cover of spruce forests decreases near the timberline, and the herb layer is also reduced due to the rocky outcrops and surfacing stones (SI, Fig. A1d). At timberline, mountain-ash (*Sorbus aucuparia*), stone pine (*Pinus cembra*), and juniper (*Juniperus communis*) shrubs (SI, Fig. A1e) are interspersed among dwarf spruce trees. Above the timberline, mugo pine (*Pinus mugo*) shrubs sometimes cover parts of the subalpine meadows (SI, Fig. A1f). This natural elevational succession of forest habitats is sometimes altered by temperature inversions or past logging and reforestation, which artificially lowered the lower limit of spruce forests. The geological substratum of the area is limestone in its south-eastern part, and crystalline schists elsewhere; thus, habitats in south-east are warmer and drier compared to similar habitats elsewhere.

Our study area is a complex mix of forest patches, some of them still in a natural or almost natural state. Logging has been forbidden in the park since its establishment, but continues beyond its borders, thus the areas with different types of human disturbance are spatially segregated. Beech and mixed forests are exploited either by clear-cut or extraction of mature trees, while spruce forests are always clear-cut, but forests at the timberline are usually kept intact. During timber extraction tree trunks are removed while branches are usually left in place, representing the majority of the coarse woody debris outside the park. By contrast, coarse woody debris in the park is represented mainly by fallen tree trunks.

Tourism is well developed in the park, especially during summer months. A paved road borders part of the southern area, facilitating the access for tourists to unpaved roads, camping grounds, chalets, and numerous trails. Some of our survey sites were established in or near touristic areas of the park. Beyond the limits of the park human disturbance is mostly by forest workers and shepherds; grazing affects mainly subalpine meadows (not surveyed).

### Small mammal trapping

All aspects of trapping and animal handling complied with EU Council Directive 86/609/EEC on experimental use of animals. Trapping within the protected area was done at the invitation of the Administration of Retezat National Park following the protocols on trapping and animal handling developed and approved by the Scientific Council of Retezat National Park.

We live-trapped small mammals using artisanal wooden box-traps (18 × 10 × 8 cm) from mid-June to early September, 2002 through 2006. Trapping sites outside the park were surveyed between 2003 and 2005. Located along four main valleys, the 26 trapping sites (Fig. 1), at elevations between 820 and 2040 m, were chosen for the best spatial coverage of elevation and forest types, 16 sites being located in the park and 10 outside it. Transects included 30–40 traps set 15 m apart along the contour lines and parallel to the closest watercourse, forest edge, road or trail, within homogenous forest habitats; ecotones were avoided. In heterogenous sites where two or three distinct types of forests were present, one transect was set in each forest type, resulting in 38 surveyed habitats—22 in the park and 16 outside it. Habitats were randomly surveyed between one and six times, resulting in 73 transects—53 in the park and 20 outside it.

Traps were baited with sunflower seeds and apple slices, but no prebaiting was done. Traps were checked in the morning and at dusk for two or three consecutive days. Because many traps were disturbed by weather, animals or people, the trapping effort differed greatly among transects, varying between 20 and 120 trap-nights per transect, resulting in a total of 3718 trap-nights. We identified captured individuals to species based on morphological traits, marked them by fur clipping, and then released each at its trapping site. Recaptures were not included in the dataset and analyses.

### Habitat characteristics

For each transect we estimated a series of habitat variables generally considered to be important for small mammal populations: percent cover of tree canopy (referred to as *tree cover* hereafter) also recorded separately for coniferous trees, percent of shrub and herbaceous layers, mean height (cm) of herbaceous layer, distance (m) to closest watercourse, river or creek (referred to as *distance to water* hereafter), and elevation (m). Tree cover was estimated by the percent of the ground where light fell directly; distance to water and elevation were measured for the trap placed at the center point of the transect. The descriptive statistics of these environmental variables are given in Table A1.

**Table 1:** Results of small mammal trapping during the 5 years of survey.

Species	Lowest elevation	Highest elevation	Number of transects	Number of individuals captured in the park	Number of individuals outside the park	Mean capture index	Maximum capture index
*Sorex araneus*	920 (820*)	2040	33	65	15	2.88	53.7
*Sorex minutus*	920	2020	6	6	0	0.27	4.5
*Sorex alpinus*	1185	1640	4	3	2	0.2	6.56
*Neomys fodiens*	1640	1640	1	1	0	0.02	2.56
*Neomys anomalus*	920	920	1	1	0	0.04	2.56
*Muscardinus avellanarius*	1150 (820*)	1650	6	2	5	0.13	3
*Glis glis*	1570	1570	1	1	0	0.01	1.1
*Myodes glareolus*	820	1840	43	155	23	5.27	58.3
*Chionomys nivalis*	1550	2040	3	5	0	0.12	3.8
*Microtus agrestis*	1300	1840	5	6	0	0.27	9
*Microtus subterraneus*	1300	1640	2	2	0	0.08	3.6
*Apodemus flavicollis*	820	2020	26	195	1	5.47	59.7
Total	820	2040	73	442	46	14.76	92.6
Trapping effort (trap-nights)				2443	1275		

*indicates data based on accidental visual observations, not included in the analyses. Trapping in the park was conducted every year but outside the park it was done mostly in 2003 and 2005 (with only one transect in 2004), both years with low abundances of the dominant rodents, hence the low number of captured individuals outside the park. Minimum capture index for each species is 0.

Soil moisture, abundance of rocks and coarse woody debris were estimated and included in analyses as ordinal variables. Soil moisture was evaluated based on the composition and structure of the vegetation and ordered as: 1—xeric, 2—mesic, 3—meso-hydric, 4—hydric. Levels of abundance of coarse woody debris and rocks were: 0—absent, 1—isolated, 2—scarce, 3—moderate, 4—abundant.

Levels of human disturbance were: 0—absent, 1—low, 2—medium, 3—high. For logging, high level of disturbance was considered for forest patches that were logged at the moment of trapping; medium level of disturbance in case of recently logged patches or those situated in the proximity of logging areas, constantly crossed by humans, horses and dogs; low disturbance level for old logging or sites situated close to the forest road, sometimes crossed by forest workers or roaming guard dogs. Human disturbance was considered absent where we found no sign of recent or old logging, some of these patches being virgin forests. For tourism, high level of disturbance was considered in case of campgrounds or habitats crossed by busy tourist trails; medium level of disturbance for habitats in the proximity of campgrounds or chalets; low disturbance level for habitats along the least intensively circulated trails. Human disturbance was considered absent in sites situated far from any tourist trail.

### Data analysis

Abundance and diversity were used as response variables. *Species abundance* was expressed as capture index, i.e., the number of captured individuals per 100 trap-nights. Species abundance was estimated per transect separately for each survey. The sum of the capture indices for all the species in a transect per survey was the *total abundance*. *Species richness* was used as the measure of assemblage diversity, expressed by the number of species captured per transect. Although some of the transects were surveyed repeatedly from year to year, because of the strong temporal fluctuations in species abundance and spatial synchrony^23^ we assumed spatial independence of samples but accounted for temporal autocorrelations, including year of survey either as random factor (in the mixed-effect models) or covariate (in the multivariate models).

To evaluate the responses to habitat characteristics of the dominant species, but also of the total abundance and species richness, we used mixed effects models in the lme4 package^24^ in the R version 3.6.1^25^. We used the Poisson generalized linear mixed models (GLMM), including as response variable the number of captured species (when analyzing species richness) and individuals (when analyzing total abundance and species abundance). Because the trapping effort differed among transects, we included it in the models as offset. We used negative binomial GLMM when overdispersion was significant. Overdispersion was tested using the check_overdispersion function in the performance package^26^. To compare the mixed models, select the best model, and test the effect of the included predictors we used the likelihood-ratio test, which assesses the goodness of fit of two competing nested models based on the ratio of their likelihoods. We used the stepwise forward selection procedure, starting from the null model (with the intercept and no explanatory variables) and adding gradually the variables that increased the most the model quality (its significance), until adding another predictor did not yield a significantly better model. The explained variation for the best model was expressed by the conditional and marginal pseudo-R squared statistics^27^, computed with the function r.squaredGLMM in the MuMIn package^28^. The marginal R-squared represents the variance explained only by the fixed part of the model, while the conditional R-squared is interpreted as the variance explained by the entire model, including both fixed and random factors.

Disturbance types were spatially segregated (tourism in the park and logging outside it); therefore, we tested the effects of disturbance caused by tourism only in the park and the effects of logging only outside the park. Because random factors must have at least five levels, when testing the effect of logging we included year as fixed factor with the levels 2003 and 2005 (we excluded the one transect from outside the park in 2004) in Poisson (or negative binomial) generalized linear models (GLM), but its effect was not significant in any model. The likelihood-ratio based pseudo-R-squared was used to estimate the explained variation for the best GLMs, calculated with the function r.squaredLR in the MuMIn package^28^. To test the effect of tourism, we used the GLMMs with year as random factor.

The effect of habitat characteristics at the community level was analysed using Canoco 5.12 software^29^. An indirect gradient analysis, the detrended correspondence analysis, was first performed to establish the length of the gradients and to summarize the variation in the small mammal community. The linear constrained ordination method—the redundancy analysis (RDA) allows to consider not only the relative abundances of species (i.e., the percent composition of species relative to the total number of individuals captured in the transect, obtained when response data are standardized by site total), which we refer to as *species composition* hereafter, but also the variation in their abundances (when response data are not standardized), which we refer to as *community abundance*. In contrast to RDA, its unimodal equivalent—the canonical correspondence analysis (CCA), is applied on standardized response data, so it can only be used to illustrate the effect of explanatory variables on species composition, but not on community abundance. Consequently, empty samples (and we had nine transects with no captures) are eliminated from CCAs but are included in RDAs. For these two reasons we chose to use RDA instead of CCA. But linear ordination methods can be used only for short ordination axes, so we excluded rare species (with < 5 occurrences) from the analyses to reduce species turnover. Response data were log-transformed by the expression y’ = log(y + 1). Interactive forward selection was applied to choose the parsimonious sets of predictors for the RDA. Probabilities were adjusted (p_adj_) to correct for the inflation of type-I error caused by multiple testing, using the false discovery rate values. This approach adjusts the *p* values in a way that limits the false discovery rate for a whole family of tests to a specified threshold (0.05 in our case)^30^. Significance of ordination axes was tested by the Monte-Carlo permutation test with 999 permutations per each test. Because of the unbalanced design, we used the model-based permutations, in which permutations were restricted to blocks defined by the covariate—the year of survey. The interactions between habitat characteristics on one hand and year and elevation, on the other hand, were used as explanatory variables, while taking all these variables as covariates, i.e. removing their main effects^30^, in order to test the possible temporal and elevational changes in the patterns of habitat use. We used the variation partitioning procedure to assess and compare the explanatory importance of the habitat characteristics and annual fluctuations, evaluating the unique (conditional) effects of the predictors and their overlap.

Because of the wide range of some explanatory variables, which makes the multiplicative relationships more meaningful than the additive ones, for the data analysis we log-transformed cover of vegetation layers (tree, shrub, and herb), height of herbaceous layer, and distance to water.

## Results

### Trapping results

During the 5 years of study we trapped 488 individuals of 12 species (Table 1). The rodents *A. flavicollis* (40.2% of the captured individuals, with the standard error—SE = 2.2%) and *M. glareolus* (36.5%, SE = 2.2%), and the shrew *S. araneus* (16.4%, SE = 1.7%) had the highest occurrence and abundance. Total abundance varied between 0 and 92.6 individuals/100 trap-nights, with a mean of 14.7 individuals/100 trap-nights (SE = 2.22). Species richness varied between zero (9 transects) and four (4 transects) species per transect, with a mean of 1.79 species (SE = 0.12). In most transects we captured only one (20 transects) or two species (25 transects), usually *M. glareolus* and *A. flavicollis* or *S. araneus*.

### Variation in total abundance and species richness in relation to habitat characteristics

The best mixed model for the number of captured species included tree cover and abundance of rocks as fixed effects and year as random effect (Table 2). Species richness increased with both tree cover and abundance of rocks. This model explained 27.3% of the variation in species richness, habitat characteristics accounting for 14%. Total abundance was best predicted by the same variables as species richness (Table 2), but having a stronger effect, explaining 21.4% of the total 72.2% of the variation in total abundance.

**Table 2:** Parameters of the fixed effects in the best GLMMs including species richness, total abundance and abundance of the three dominant species as response variables, habitat characteristics as fixed effects and year as random factor.

Variable	Coefficient	Standard error	χ^2^	*p*	Marginal pseudo-R^2^	Conditional pseudo-R^2^
Species richness					0.14	0.273
Intercept	0.441	0.466				
Tree cover	0.291	0.091	8.08	0.004		
Rocks	0.263	0.112	5.414	0.019		
Total abundance					0.214	0.722
Intercept	-0.611	0.519				
Tree cover	0.379	0.075	20.739	< 0.001		
Rocks	0.301	0.088	10.981	< 0.001		
*Apodemus flavicollis*					0.082	0.955
Intercept	-0.072	0.896				
Shrub cover	-0.291	0.062	21.14	< 0.001		
Moisture	0.242	0.11	4.34	0.037		
Distance to water	-0.112	0.04	7.97	0.004		
*Myodes glareolus*					0.417	0.627
Intercept	-2.104	0.771				
Tree Cover	0.699	0.153	22.178	< 0.001		
Moisture	-0.535	0.157	9.778	0.001		
Rocks	0.466	0.158	8.166	0.004		
*Sorex araneus*					0.427	0.524
Intercept	-4.291	1.32				
Tree cover	0.736	0.29	10.291	0.001		
Rocks	0.497	0.176	7.848	0.005		

Significance of predictors was tested using the likelihood-ratio test. Marginal pseudo-R^2^ represents the variance explained only by the fixed part of the model and conditional pseudo-R^2^ represents the variance explained by the entire model.

### Habitat use by the dominant species

Abundance of *A. flavicollis* was best predicted by shrub cover, moisture and distance to water, with its numbers decreasing in dry habitats with dense shrubby undergrowth far from watercourses. However, most variation in the abundance of this species was associated with year-to-year fluctuations; only 8.2% of explained variation could be attributed to habitat characteristics (Table 2). Abundance of *M. glareolus* increased in dry, rocky, and dense canopy forests (Table 2); habitat characteristics explained 41.7% of the total 62.7% variation explained by the mixed model. In *S. araneus* habitat characteristics (42.7% explained variation) were even more important than year (9.7% explained variation) for determining population densities. Abundance of *S. araneus* was higher in rocky habitats with dense tree canopy layer (Table 2).

### Effects of human disturbance on small mammals

Intensity of human disturbance by logging had a significant negative effect on species richness (χ^2^ = 11.42, *p* < 0.001, pseudo-R^2^ = 45.2%), total abundance (χ^2^ = 21.57, *p* < 0.001, pseudo-R^2^ = 67.8%), and the abundance of *S. araneus* (χ^2^ = 9.86, *p* = 0.001, pseudo-R^2^ = 40.5%), but not on the abundance of *M. glareolus*. During the survey in areas affected by logging, *A. flavicollis* had very low population densities so that we captured only one individual, making any analysis impossible.

Intensity of human disturbance by tourism had only a weak effect on the abundance of small mammal species, being significant only for *M. glareolus* (χ^2^ = 4.52, *p* = 0.033, marginal pseudo-R^2^ = 11.3%), which had higher abundances in habitats with no tourist flow. Trapping success of *A. flavicollis* was higher in the more disturbed sites, but when accounting for multiannual fluctuations, although the effect of disturbance was positive, it was not significant.

### Responses of small mammal communities to habitat characteristics

The habitat characteristics that we evaluated were significant predictors of small mammal assemblages (pseudo-F = 3.0, *p* = 0.001), explaining 34.5% (23% adjusted) of the partial variation in the community abundance, i.e., after we removed the effect of year.

**Figure 2:**
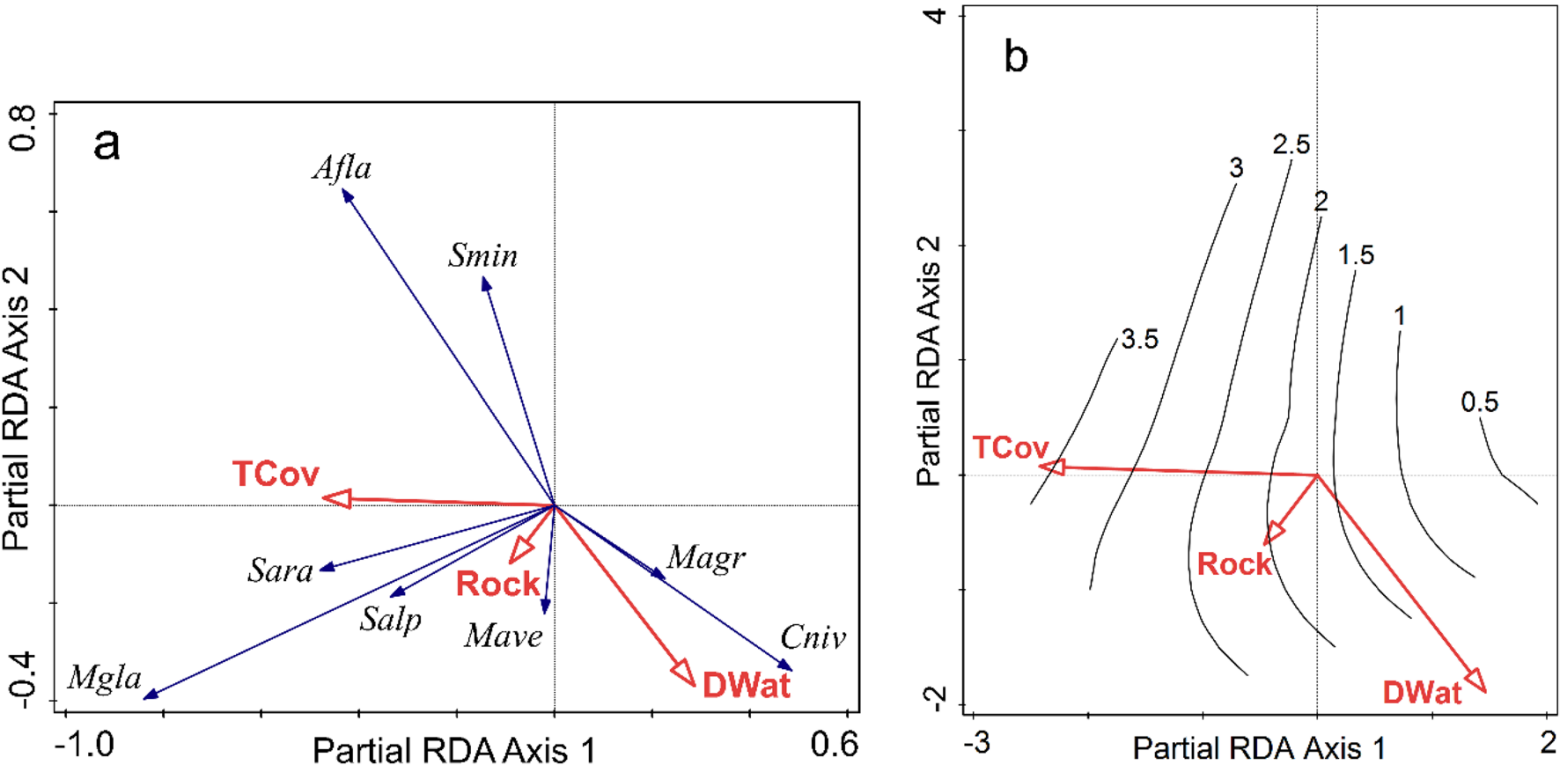
(**a**) Species—habitat biplot diagram from partial RDA (year included as covariate) summarising the effect of tree cover (TCov), distance to water (DWat) and rocks (Rock) on the community abundance (response data were not standardised by site). The codes for species are given by the initial of genus and first three letters of species name. The length of the arrows representing the predictors is given by the strength of their correlation with the first two ordination axes (indicated by the projection of the arrows on the two axes). The angle between arrows indicates the correlation between individual variables. The angle between species arrows indicates the correlation between the capture index of species (positive when the angle is sharp). The length of the arrow is a measure of fit for the species. (**b**) contour plot of species richness within the ordination space of the first two axes of partial RDA, based on a fitted loess model.

Tree cover, distance to water and elevation were significant predictors of community abundance when considered separately. In addition, soil moisture and rocks had marginally significant effects when controlling for type-I error inflation, using the adjusted *p* value (Table 3). These habitat characteristics had mostly non-overlapping simple effects; thus, they had also significant conditional effects, except for elevation and soil moisture. After accounting for the effects of the other variables, elevation and moisture did not explain the residual variation in community abundance, their conditional effects being not significant (Table 3). Thus, the most parsimonious set of predictors included tree cover, distance to water, and abundance of rocks, which together had a significant effect (pseudo-F = 7.4, *p* = 0.001), and explained 25.7% (22.2% adjusted) of the residual variation in community abundance after removing the effect of multiannual fluctuations. The first constrained ordination axis (explaining 15.9% of the variation, pseudo-F = 4.0, *p* = 0.001) was defined mainly by tree cover and to a lesser extent by distance to water and abundance of rocks. Along this ordination axis the dominant species were positively correlated with the tree cover, showing a positive response to this habitat characteristic, strongest in *M. glareolus*. Distance to water and abundance of rocks were the contributors to the second ordination axis (explaining 6.8%, pseudo-F = 2.8, *p* = 0.003). The abundance of *S. minutus* and *A. flavicollis* was higher in habitats close to watercourses, irrespective of the extent of rocky substratum, while *M. glareolus, S. araneus,* and *S. alpinus* were more abundant in habitats with abundant rocky outcrops, regardless of the distance to water; *M. agrestis* and *C. nivalis* increased in abundance with increased distance to water (Fig. 2a). These three predictors explained 49.8% of the variation in the species richness in the ordination space (pseudo-F = 5.4, *p* = 0.006); richness increased with tree cover and less so with the abundance of rocks, but was independent of the distance to water (Fig. 2b). Tree cover remained the best predictor even when we excluded shrubby sites above the timberline from the analysis. Response of community abundance to habitat did not change among years in time or along the elevational gradient, so the interaction between either year or elevation and these predictors was not significant.

**Table 3:** Simple and conditional effects of the habitat variables on the community abundance and species composition of small mammals in the study area.

Variable	Community abundance								Species composition							
	Simple term effects				Conditional term effects				Simple term effects				Conditional term effects			
	Explains %	pseudo-F	*p*	p_adj_	Explains %	pseudo-F	*p*	p_adj_	Explains %	pseudo-F	*p*	p_adj_	Explains %	pseudo-F	*p*	p_adj_
Tree cover	**13.9**	**10.6**	**0.001**	**0.007**	**13.9**	**10.6**	**0.001**	**0.005**	**4.5**	**2.7**	**0.01**	**0.028**	**4.7**	**3.2**	**0.011**	**0.037**
Shrub cover	3.2	2.2	0.058	0.097	0.7	0.6	0.635	0.635	1.6	1	0.387	0.391	0.4	0.3	0.945	0.945
Herbaceous cover	1.6	1.1	0.353	0.353	1.3	1.1	0.306	0.612	1.7	1	0.391	0.391	3.8	2.7	0.029	0.073
Herbaceous height	1.3	0.9	0.318	0.353	0.8	0.7	0.506	0.616	**5.4**	**3.3**	**0.019**	**0.038**	0.3	0.2	0.932	0.945
Conifers	3.1	2.1	0.104	0.149	1	0.9	0.438	0.616	4.5	2.7	0.054	0.090	1.1	0.8	0.561	0.701
Coarse woody debris	2.4	1.6	0.148	0.185	0.8	0.7	0.554	0.616	2.9	1.7	0.154	0.198	2.3	1.6	0.22	0.380
Rocks	3.6	2.5	0.04	0.080	**5.7**	**4.9**	**0.001**	**0.005**	2.9	1.7	0.158	0.198	1.6	1.2	0.372	0.531
Moisture	5	3.4	0.023	0.058	3	2.7	0.041	0.103	**8.1**	**5.1**	**0.003**	**0.015**	**8.1**	**5.1**	**0.001**	**0.010**
Distance to water	**8.7**	**6.3**	**0.002**	**0.007**	**6.2**	**5**	**0.002**	**0.007**	**8.1**	**5.1**	**0.002**	**0.015**	1.9	1.4	0.228	0.380
Elevation	**7.6**	**5.4**	**0.002**	**0.007**	1.2	1	0.377	0.616	**5.6**	**3.5**	**0.011**	**0.028**	**6.1**	**4**	**0.003**	**0.015**

Values of the explained variation (Explains %), pseudo-F, significance (*p*) and False discovery rate (p_adj_) are presented. In bold are predictors with significant effects (p_adj_ < 0.05).

**Figure 3:**
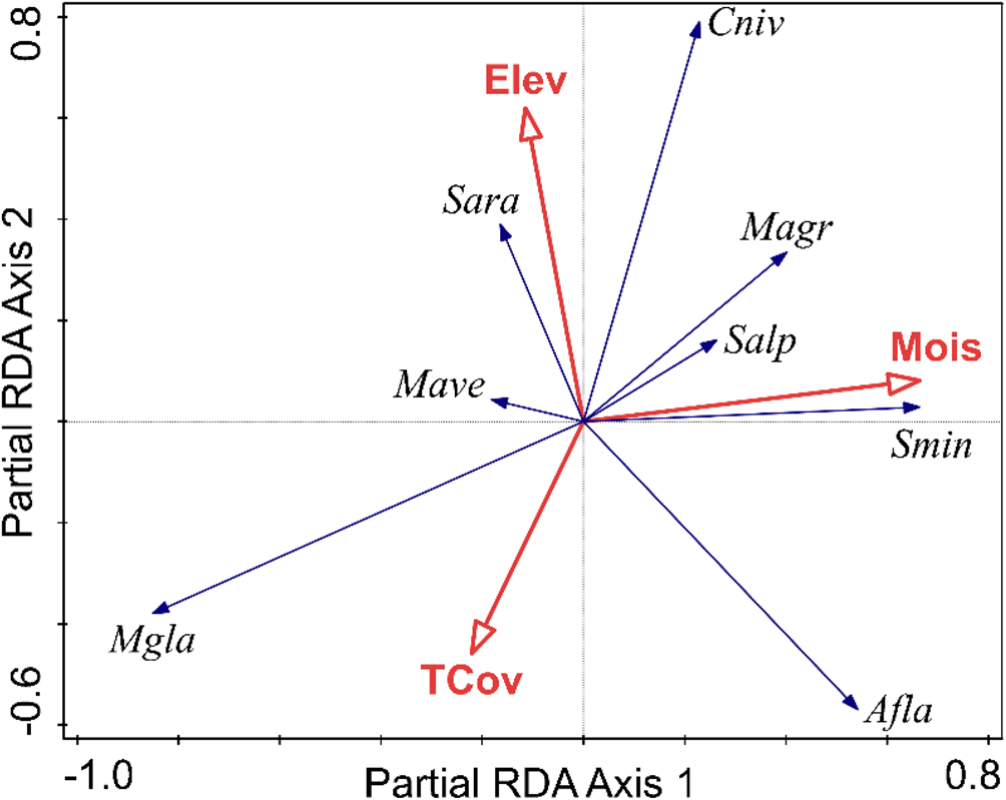
Species—habitat biplot diagram from partial RDA (year included as covariate) summarising the effects of tree cover (TCov), soil moisture (Mois) and elevation (Elev) on the species composition (response data were standardised by site total). The codes for species are given by the initial of genus and first three letters of species name. The length of the arrows representing the predictors is given by the strength of their correlation with the first two ordination axes (indicated by the projection of the arrows on the two axes). The angle between arrows indicates the correlation between individual variables. The angle between species arrows indicates the correlation between the relative abundances of species (positive when the angle is sharp). The length of the arrow is a measure of fit for the species.

In comparison to community abundance, species composition was slightly less well predicted by habitat characteristics; 30.2% (15.9% adjusted) of the partial variation could be accounted for by all habitat characteristics considered together (pseudo-F = 2.1, *p* = 0.002). Moisture, distance to water, height of herbaceous cover, elevation, and tree cover had significant simple effects; the effect of proportion of conifers in the canopy was only marginally significant (Table 3). However, the effect of these predictors on species composition was partially overlapping, so that only elevation, moisture and tree cover maintained their significance when accounting for the effect of other predictors. Together these variables explained 18.8% (14.5% adjusted) of the partial variation in species composition (pseudo-F = 4.3, *p* = 0.001). Relative preferences for the considered habitat characteristics were completely different among the three dominant species (Fig. 3). Relative abundance of *M. glareolus* increased in drier habitats, with dense canopy cover, regardless of elevation. *A. flavicollis* had higher relative abundances at lower elevations irrespective of the tree cover, while *S. araneus* preferred high elevation habitats with sparse tree cover. The other two species of *Sorex* and *M. agrestis* increased their relative abundance in damp habitats. *C. nivalis* reached the highest ratio in the communities of high elevation habitats, above or close to the timberline, with no or little tree cover. The response of *M. avellanarius* was less pronounced (Fig. 3).

The effect of moisture, elevation, and tree cover on relative abundances varied significantly in time, when we considered only the three dominant species (SI, Fig. A2). The interaction between each of these predictors and year explained 33% (13% adjusted) of the residual variation in species composition (pseudo-F = 1.6, *p* = 0.023), with the dominant species having completely divergent trends.

### Separate effects of habitat characteristics and year

In the two groups (habitat characteristics and year) variation partitioning, when analysing the unique (conditional) and overlapping effects of the variable groups on community abundance, year had the strongest unique effect. It explained 34.2% (32.3% adjusted, pseudo-F = 10.7, *p* = 0.001) of the total variation, the selected habitat variables (tree cover, rocks, distance to water) explained 17.8% (16.3% adjusted, pseudo-F = 7.4, *p* = 0.001) (mean square was 0.09 for year and 0.06 for habitat) and there was no overlap between the effects of the two groups of predictors.

Similar results were obtained for species composition, where year explained 26.7% (23.6% adjusted, pseudo-F = 6.2, *p* = 0.001), the habitat variables (tree cover, moisture, and elevation) explained 14% (11.5% adjusted, pseudo-F = 4.3, *p* = 0.001) with no overlap between the effects of the two variable groups.

## Discussion

We surveyed small mammal communities in a montane area along the elevational gradient in relation to habitat characteristics and human impact, this study being the first to assess habitat use by small mammals in the Southern Carpathians.

Compared to a similar study conducted in the Eastern Tatra Mountains^31^, the species richness (12 species captured) was lower in our survey; part of the reason could be that the North Carpathian endemic *Microtus tatricus* and the boreal species *Sicista betulina* are absent in our study area, which is beyond the limits of their geographical distribution. Species composition of small mammals was overall comparable to those reported for forested areas of Northern Carpathians^17,32,33^, although a high variability, both spatial and temporal, in the number and abundance of species characterized all surveyed communities. Although *A. flavicollis* was seldom captured in 2003 and 2005 and only at low elevations^23^, overall it, together with *M. glareolus,* dominated the small mammal community, representing over 75% of the captured individuals (Table 1). This is the common pattern of small mammal communities in temperate zones, i.e., to be dominated by two species, usually rodents^34–36^. *M. glareolus* and *A. flavicollis* are the dominant species in most forests of central and eastern Europe^32,33,37,38^, with one or the other being more numerous depending on habitat conditions and geographic position^35^. *M. glareolus* and *A. flavicollis* were also found to remain dominant in small-sized clearings^39^.

Box-trapping results for shrews are often considered underestimates because of their small size^40^ and because seed baits are not attractive to them^41^. However, during our survey *S. araneus* had wider distribution than *A. flavicollis*; we captured it in low numbers in a large number of trapping sites, having the highest ratio between occurrence (45.2%) and relative abundance (16%) of all small mammal species (Table 1). *S. araneus* was higher in abundance in our research area in comparison to both natural and planted montane forests in Northern Carpathians^17,32,33^, possibly as an effect of the long-term conservation practices in the national park.

Besides the three dominant species and *S. minutus*, all the other captured species are of regional conservation interest, being included in the Red Book of Vertebrates from Romania^42^, which highlights the conservational value of this landscape.

Small mammals showed significant responses to habitat characteristics at population and community levels, regardless of the metrics considered. Tree cover was an important predictor for small mammal communities (Table 2, Table 3). Increased tree cover limits light available for understory plants, reducing habitat structure^43^, hence the usually negative correlation between canopy cover and both shrub and herbaceous cover. The reduced vegetation complexity of closed-canopy forests may limit resources important to small mammals. Most studies show that forests with a greater percentage of tree cover harbour less abundant small mammal communities^44^. In the Sierra Nevada mountains in North America, small mammals showed a limited response to canopy thinning, reflecting the generalist habits of the common species in those forests, which may be a legacy of more than a century of human impacts generating a process of biotic homogenization via differential success of some native species over the others^45^. In Europe, there is a legacy of much longer human impacts, thus common forest species should have even more generalist habits. However, in our research area tree cover was positively correlated with all parameters, except for the abundance of *A. flavicollis*, which did not significantly respond to it (Table 2). The small mammal fauna in our study area is a primarily forest fauna, with dominant species responding negatively to the decrease in tree canopy cover, even when this means an increase in the understory cover and complexity. The response to tree cover was strongest in *M. glareolus* (Fig. 2a, Fig. 3). In boreal forests of Scandinavia tall vegetation and structural heterogeneity of trapping stations positively influenced the total abundance of this species^15^. This may mean that there is an important geographic variability in the ecological behavior of *M. glareolus*. There are differences in the habitat preferences not only along the latitudinal gradient^15,35,46,47^ but also on elevation. At the foothills of Southern Carpathians *M. glareolus* is limited mainly to forest edges and riparian forests with tall hygrophilous vegetation^48^. During this study we did not find a significant effect of the interaction between elevation and tree cover, probably because of the relatively short elevational gradient (of 1200 m), which did not include lowland forests outside the ecological optimum of *M. glareolus*. The short gradient may also explain the lack of response by *M. glareolus,* both as absolute and relative abundance (Fig. 3) to elevation itself, although this species is known to increase in density towards the north and at higher elevations^35^.

Although shrub cover is an important element of vegetation structure, and one which increases its complexity, it had a significant effect only on the abundance of *A. flavicollis*. In opposition to our expectations, we found increased abundances of *A. flavicollis* in forests with little or no shrub layer (Table 2). In forests, shrubs may serve as shelter for mice against physical disturbances such as soil compaction, trampling or rooting^49^, although some studies failed to find evidence for this^50^. A positive effect of cover and height of shrub layer was also found on the abundance of *A. flavicollis* in the Northern Carpathians in forest clearings^51^. However, besides the positive effects of greater vegetation complexity and increased availability of food and shelter resources, the shrub layer also reduces visibility and hinders rapid movement, so that mobile species such as mice, which rely on running rather than hiding to escape predation, are exposed to higher predation risk in habitats with dense undergrowth.

The feature related to habitat heterogeneity to which small mammals responded positively in our study area was the abundance of rocks (Fig. 2a, Table 2). Rocky outcrops and large boulders are stable elements of the landscape that enhance the availability of shelters and refuges providing hard protection for nest sites^50^. Some species that do not burrow are dependent on rocks for shelter, occurring only in rocky sites. Among these is *C. nivalis*, but the small number of captured individuals did not allow testing its habitat use.

Unlike rocks, woody debris is more ephemeral, and apparently it was less valued as a shelter resource (Table 3). Many studies show the importance of coarse woody debris as a quantitative habitat feature for forest small mammals^44^; their value increases in the late decay stages^52^. Woody debris in mid-to-late decay state is often a suitable substrate for lichen and fungi, and can support a rich insect fauna^53^, all potential foods for omnivorous rodents and shrews. In our research area the sites with the largest amounts of coarse woody debris were those recently logged, so availability of food resources for small mammals was not optimal.

Soil moisture, which has a very strong effect on the primary productivity and vegetation diversity, may also have an important role in the habitat selection, with various effects on small mammal populations. In our study area the two dominant rodents had opposite responses to soil moisture, with *M. glareolus* showing a strong preference for dry habitats (Fig. 3, Table 2), in contrast to its response to moisture in other parts of its distribution. At the southern limit of its geographical distribution^35^ or at the limit of its elevational distribution^48^, *M. glareolus* is usually confined to damp habitats, but there it does not develop abundant populations, with *Apodemus* species usually dominating the small mammal community. In the northern part of its distribution, where *Apodemus* species are absent, *M. glareolus* also shows a preference for moist woodlands^54^. We may thus infer that the response of *M. glareolus* to soil moisture is modulated by the interaction with mice species, in our case *A. flavicollis*. This conjecture is also supported by the fact that moisture did not significantly affect community abundance, only species composition (Table 3). Other studies have also reported conflicting results of the role of soil moisture for *A. flavicollis*. For example, it was one of the most important factors influencing population dynamics of *A. flavicollis* in a beech forest in northern Germany^55^ but it did not predict its distribution in Britain^56^.

Sites closer to watercourses are damper, so an overlap of the effect of the two variables—moisture and distance to water—would be expected. However, the significant negative effect of distance to water on the abundance of *A. flavicollis* also had a component that was independent of soil moisture (Table 2), and this may have a spatial significance. The increased abundance of *A. flavicollis* in sites close to watercourses could be explained by a potential fence effect that these may exert on small mammal populations. River banks are linear habitats bordered on one side by a physical barrier, more or less penetrable depending on the local habitat morphology. Linear habitats with favourable conditions sometimes shelter rodent populations at densities much higher than those in wide habitats, although the underlying mechanism, involving probably territoriality and dispersal, is not yet understood^57^. In our research area, river banks were important for *A. flavicollis* especially in low abundance years, when we captured this species exclusively here and only at low elevations, suggesting that besides a source of habitat heterogeneity watercourses may be involved also in the spatial dynamics of populations, with their banks being used as routes for dispersal.

Neither species richness nor species abundance changed along the elevational gradient in our research area when also considering yearly fluctuations and habitat characteristics (Table 2), and our result is in contradiction with the pattern frequently described for mountains worldwide^58,59^, including the Eastern Tatras^31^, which shows a reduced species richness with the increase in elevation. But on the other hand, we found species composition to be affected by elevation, with *A. flavicollis* responding negatively and *S. araneus* positively. The thermophilous character of *A. flavicollis* is more evident in the Northern Carpathians, where this species was found only up to 1328 m, well below the timberline^31^. But as latitude compensates for elevation, at least in part, in our research area *A. flavicollis* was found along the entire elevational gradient, up to above 2000 m (Table 1), beyond the timberline, in the subalpine shrubs, perhaps as a result of its lack of preference for the tree cover. *S. araneus* had a similarly wide elevational distribution and, unlike *A. flavicollis*, it was captured at high elevations also in low abundance years^23^. This result supports the classification of *S. araneus* as a habitat generalist. In contrast to these species, *M. glareolus* was only once captured in the shrubs beyond the timberline, suggesting that in our study area this vole avoids habitats with no tree layer. This may also be because the subalpine sites that we surveyed were heterogenous, with relatively small patches of shrubs separated by open meadows, areas avoided by *M. glareolus*.

Logging is the main human activity causing disturbance of forests. In our study area only selective logging was recent, while older clearcuts were already reforested. The overall impact was negative and significant on species richness and total abundance, as well as on the abundance of *S. araneus*. The sensitivity of *S. araneus* to logging may be one cause of its increased abundance at higher elevations, as in the study area recent timber exploitation was concentrated at low elevations (mostly in mixed forests). Although we did not find a significant response of *M. glareolus* to logging, other studies revealed that this species is influenced by habitat alterations caused by logging^15^ but also by the inter- and intraspecific competition, which is considered by some investigators to be the main mechanism causing the decline of vole populations in harvested forests^60^. We learned that timber exploitation caused a drastic reduction of the small mammal populations in the disturbed area, to the point where no animal was captured during a trapping session, with the neighbouring habitats being also affected. However, since habitat changes were not substantial, timber extraction had a relatively short time impact on the small mammals, and the year following logging the community structure resembled that of undisturbed areas. This suggests that selective logging with the extraction of a relatively small amount of timber affects small mammals rather by direct disturbance than by changes in habitat characteristics. The influence of logging on species of conservation interest, such as the mostly arboreal *M. avellanarius* and the rare *S. alpinus*, still needs to be evaluated. The main effect of logging is the decrease in canopy cover or its complete removal in case of clearcuts. But there are also other effects, such as degradation of shrub and herbaceous layers, soil compaction and erosion, and also direct disturbance involving presence of humans and sometimes domestic animals (in the research area logged trees were removed by horses and watch dogs usually roamed the logged forest patches and their surroundings), noise and soil vibrations. Following reduction of canopy cover, improvement in light conditions cause development of understory and decrease of soil moisture, affecting the abundance and composition of animal communities. Most studies on the influence of forest management on small mammals in Europe have focused mainly on clearcutting, one of the most common methods of forest harvest, and have revealed a positive effect on most analyzed small mammals, which can be attributed to an increase in forb and grass cover in the harvested areas^61^. In managed forest in Czech Republic it was found that the practice of felling within relatively small-sized clearings may help preserve the diversity of small mammal community^39^. However, the observed positive effect of clearcuts may be a biased result caused by the fact that most surveyed sites were in homogenous conifer plantations, a low-quality habitat for small mammals^61^.

We found that tourism had less impact on small mammals compared to logging, with *M. glareolus* showing the only significant negative response. Tourism may also represent an additional source of food for the small mammal species that tolerate the presence of humans, such as *A. flavicollis*, which we found on campgounds. Touristic buildings may also represent important daily or hibernation shelters for some rodents, such as *Glis glis*, which we observed in autumn in a chalet. In contrast to logging, the effect of tourism on small mammals has been less researched and most such studies have focused on winter sports resorts and mainly on the impact of ski-run development, which involves substantial alteration of forest habitat, sometimes with a significant change in small mammal communities^62^. In case of ecotourism, damage to the vegetation and soil compaction that result from trampling during tourist season is only local and temporary, thus the regeneration of soil fauna and vegetation is possible^63^, hence the weaker effect of ecotourism on small mammals.

Habitat characteristics had a stronger influence on community abundance than on species composition (Table 3), suggesting that, being primarily forest dwellers, the small mammal species in our study area have somewhat similar responses, especially towards tree cover, but they also show some differentiation, which is reflected by the divergent responses of *A. flavicollis, M. glareolus,* and *S. araneus* in their relative abundances in the community. The differences in the relative habitat use, along with the divergent dietary niche, enables their coexistence as dominant species, exploiting the same wide range of habitat resources.

In conclusion, habitat use by small mammals in the continuous forest landscape in the Southern Carpathians was overall similar to that reported from the Northern Carpathians, with some notable differences related to recent and historical forest management practices and to latitude. Variation partitioning showed that yearly fluctuations were more important than habitat selection in shaping community composition. Temporal variations eclipsed the effects of habitat selection and elevational gradient, temporal fluctuations in community abundance and species composition having higher amplitudes than spatial variations. Relative habitat use by most species also changed among years. Thus, our results suggest that ignoring the time dimension of habitat selection may lead to the inability to comprehend the forces and processes that structure small mammal communities.

## Supplementary information

Supplementary Information.

## Data Availability

The raw data used for this study are publicly available in the Dryad data repository^64^.
