# Supplementary material for: Responses of small mammals to habitat characteristics in Southern Carpathian forests
Source: Sci Rep. 2021 Jun 8;11:12031. doi: 10.1038/s41598-021-91488-6 (PMC8187625; doi:10.1038/s41598-021-91488-6)
Supplement: Supplementary file 1 — Supplementary Information. [file 41598_2021_91488_MOESM1_ESM.pdf]

Scientific Reports

Responses of small mammals to habitat characteristics in South Carpathian forests

Ana Maria Benedek, Ioan Sîrbu, Anamaria Lazăr

Corresponding author: Ana Maria Benedek, Lucian Blaga University of Sibiu, Faculty of Sciences, Applied Ecology Research Center, 5-7 Raţiu Street, 550012 Sibiu, Romania, ana.benedek@ulbsibiu.ro, benedek_ana@yahoo.com,

Supplementary Information

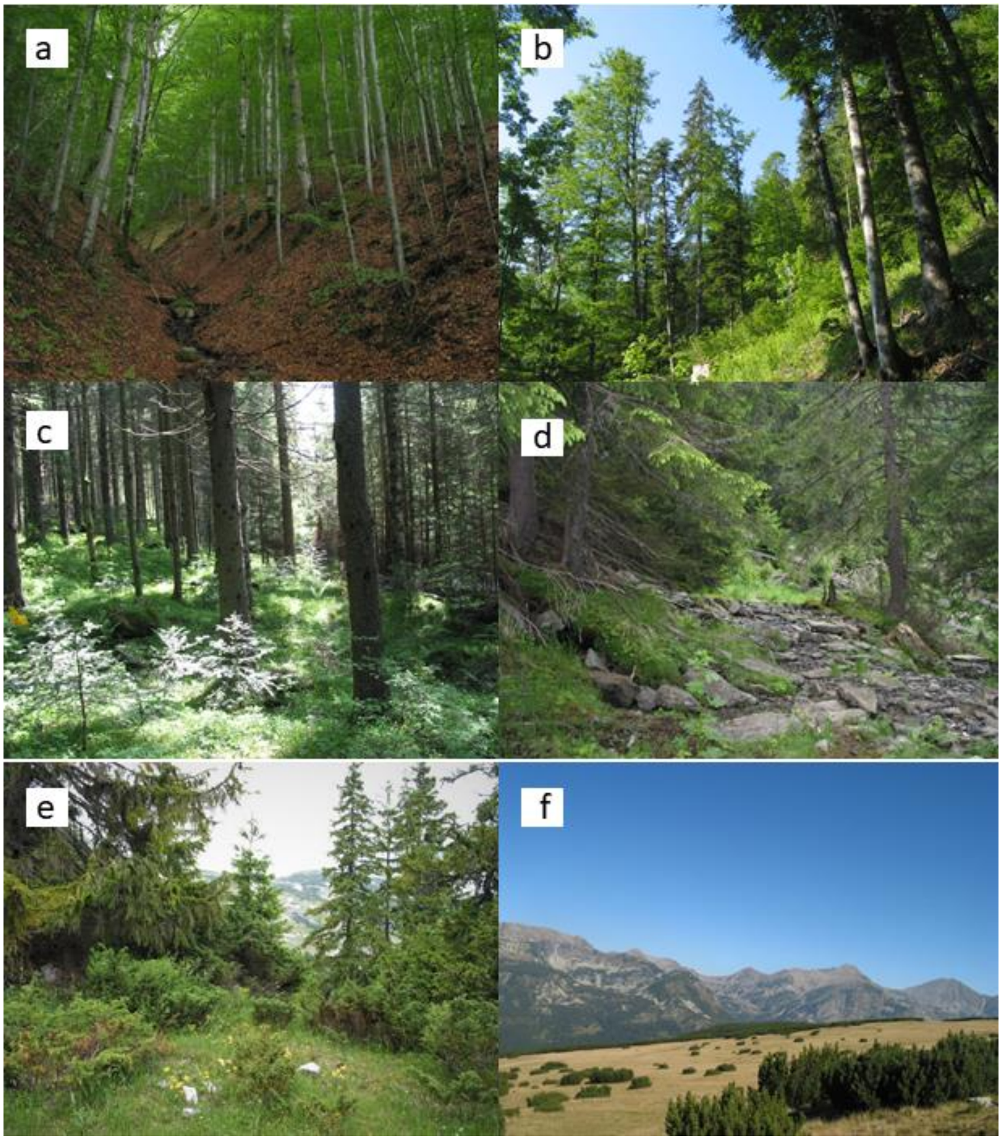

Fig. A1 Typical habitats along the altitudinal gradient in the study area: a – homogenous, dense canopy beech forest with sparse shrub and herbaceous layers; b – mixed forest dominated by beech, Norway spruce and silver fir with rich shrub and herbaceous layers, recenty logged; c – dense Norway spruce forest with numerous saplings and rich undergrowth dominated by blueberry bushes; d – sparse spruce forest with hygrophilous vegetation and numerous rocky outcrops and stones; e – sparse spruce forest at timberline with dense juniper shrubs; f – sparse stone pine shrubs in the subalpine meadows, above the timberline

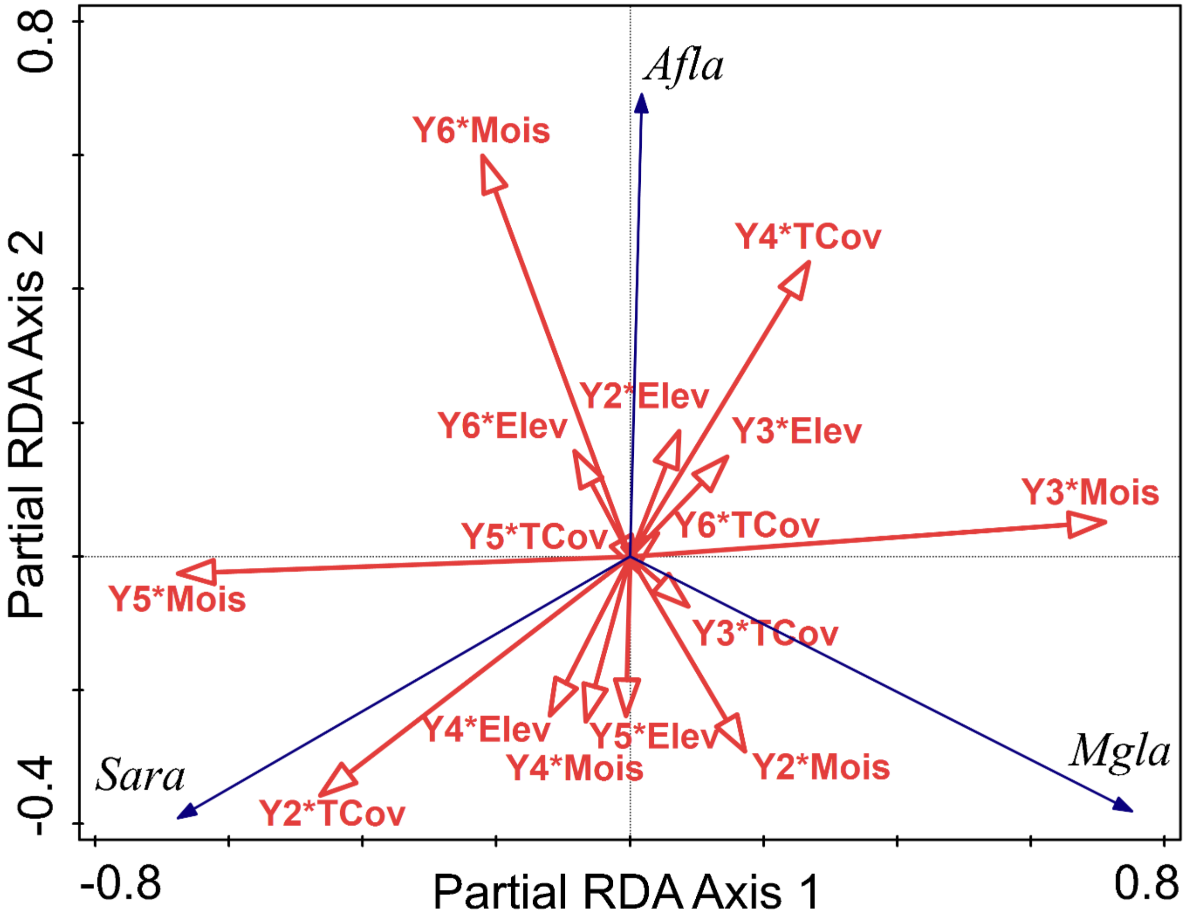

Fig. A2 Biplot diagram from partial redundancy analysis (RDA) summarising the effect of the interaction between year (Y) and the significant habitat variables - tree cover (TCov), soil moisture (Mois) and elevation (Elev) - on the relative abundance of the three dominant species (response data were standardised by site). The symbol * stands for interaction. The main effects of the predictors were included as covariates. The codes for species are: Afla – Apodemus flavicollis (yellow-necked mouse), Mgla – Myodes glareolus (bank vole), Sara – Sorex araneus (common shrew). The codes for years are: 2 – 2002, 3 – 2003, 4 – 2004, 5-2005, 6 – 2006

Table A1 Descriptive statistics of the quantitative environmental variables in the surveyed habitats. The lower and upper 95% confidence limits of the mean are designated as lower c.l. and upper c.l., SE is the standard error of the mean

	Distance to water (m)	Tree cover (%)	Shrub cover (%)	Herbaceous cover (%)	Herbaceous height (cm)	Conifers in the tree cover (%)	Elevation (m)
Mean	137.9	65.14	18.29	45.75	29.24	72.47	1382
Lower c.l.	86.8	58.66	14.41	39.01	25.95	63.92	1311.5
Upper c.l.	189.1	71.62	22.16	52.50	32.54	81	1452.6
SE	25.7	3.25	1.94	3.38	1.65	4.29	35.4
